# A parsimonious approach for screening moderate-to-profound hearing loss in a community-dwelling geriatric population based on a decision tree analysis

**DOI:** 10.1186/s12877-019-1232-x

**Published:** 2019-08-07

**Authors:** Min Zhang, Zhaori Bi, Xinping Fu, Jiaofeng Wang, Qingwei Ruan, Chao Zhao, Jirong Duan, Xuan Zeng, Dian Zhou, Jie Chen, Zhijun Bao

**Affiliations:** 1 Shanghai Key Laboratory of Clinical Geriatric Medicine, Huadong Hospital, Fudan University, 221 West Yan’an Road, Shanghai, China; 2 National Clinical Research Center for Aging and Medicine, Huashan Hospital, Fudan University, Shanghai, China; 3 Speech and Hearing Rehabilitation Department, Punan Hospital, Fudan University, Shanghai, China; 4 The State Key Laboratory of ASIC & System, Department of Microelectronics, Fudan University, Shanghai, China; 5 Department of Electrical Engineering, University of Texas at Dallas, Richardson, TX USA

**Keywords:** Community-dwelling geriatrics, Hearing screening, Decision tree

## Abstract

**Background:**

Hearing loss is one of the most common modifiable factors associated with cognitive and functional decline in geriatric populations. An accurate, easy-to-apply, and inexpensive hearing screening method is needed to detect hearing loss in community-dwelling elderly people, intervene early and reduce the negative consequences and burden of untreated hearing loss on individuals, families and society. However, available hearing screening tools do not adequately meet the need for large-scale geriatric hearing detection due to several barriers, including time, personnel training and equipment costs. This study aimed to propose an efficient method that could potentially satisfy this need.

**Methods:**

In total, 1793 participants (≥60 years) were recruited to undertake a standard audiometric air conduction pure tone test at 4 frequencies (0.5–4 kHz). Audiometric data from one community were used to train the decision tree model and generate a pure tone screening rule to classify people with or without moderate or more serious hearing impairment. Audiometric data from another community were used to validate the tree model.

**Results:**

In the decision tree analysis, 2 kHz and 0.5 kHz were found to be the most important frequencies for hearing severity classification. The tree model suggested a simple two-step screening procedure in which a 42 dB HL tone at 2 kHz is presented first, followed by a 47 dB HL tone at 0.5 kHz, depending on the individual’s response to the first tone. This approach achieved an accuracy of 91.20% (91.92%), a sensitivity of 95.35% (93.50%) and a specificity of 86.85% (90.56%) in the training dataset (testing dataset).

**Conclusions:**

A simple two-step screening procedure using the two tones (2 kHz and 0.5 kHz) selected by the decision tree analysis can be applied to screen moderate-to-profound hearing loss in a community-based geriatric population in Shanghai. The decision tree analysis is useful in determining the optimal hearing screening criteria for local elderly populations. Implanting the pair of tones into a well-calibrated sound generator may create a simple, practical and time-efficient screening tool with high accuracy that is readily available at healthcare centers of all levels, thereby facilitating the initiation of extensive nationwide hearing screening in older adults.

## Background

Age-related hearing loss adds a significant burden to individuals and society. Compared to their normal hearing peers, individuals with hearing loss are at significantly greater risk of incident dementia [1–3], falls [4, 5], depression [6, 7], social isolation [8], loss of independence [9], early retirement and unemployment [10], and hospitalization [11]. Hearing loss ranked as the 13th highest contributor to the global burden of disease in 2002 and is projected to be the 9th leading contributor worldwide in 2030 [12]. Excess medical costs resulting from hearing impairment ranged from $3.3 to $12.8 billion in the United States [13, 14] and were $11.75 billion in Australia [15]. However, the adverse consequences of untreated hearing loss are still largely underestimated by society, by senior citizens and by health care professionals [16].

The existing literature supports the hypothesis that treating hearing loss is effective in reducing the aforementioned adverse consequences. Nonetheless, moving towards early treatment requires the early identification of individuals with hearing loss. Hearing impairment is highly prevalent in older adults, affecting 33% of persons over the age of 50 years, 45% of persons over the age of 60 years [17] and 63.1% of the population aged 70 years or older [3]. Despite this, fewer than one-fifth of adults with hearing loss seek or obtain any form of treatment [18]. Age-related hearing loss is severely underrecognized and undertreated.

The gold standard for estimating hearing impairment is clinical pure tone audiometry administered by trained audiologists [19]; however, this method is not feasible for large-scale, population-based epidemiological screening projects because it requires access to high-cost audiological equipment and trained personnel. The availability of an effective and sustainable hearing loss screening strategy that is fast, accurate, and easy to use is crucial and a prerequisite for the implementation of effective intervention programs, especially in developing countries with high population density and few hearing care resources.

A number of time-efficient hearing screening strategies have been proposed over the past few years. Portable screeners, such as Audioscope [20], screen hearing by delivering 4 tones of different frequencies (0.5, 1, 2, and 4 kHz) at approximately 40 dB. Although this approach provides 90% sensitivity and requires minimal training to administer, it lacks cost-effectiveness because the equipment is not affordable for primary care clinics at the community level. The hearing handicap inventory for the elderly screening (HHIE-S) questionnaire [21, 22] offers an economic option for hearing screening and can be completed within a few minutes with reasonable sensitivity (74.6%~ 84.5%) [19, 23]. However, self-reporting of hearing loss is strongly impacted by individuals’ denial or non-acceptance of hearing loss and, as a result, consistently underestimates the actual prevalence of disability [23]. A growing number of application-based hearing tests have provided an accessible and free hearing screening approach by simulating the standard audiometric testing procedure via the internet and personal smart devices [24]. However, the participation rate is low, as these application programs require initiation by older adults, whose motivation to use new technology is low. In addition, the accuracy of these application-based tests is mixed in the literature [25].

Despite the intensive effort to promote hearing screening in community-based geriatric populations, none of the currently available screening instruments have been systematically implemented, and their correlation to clinical audiometric tests varies. To date, no studies have shown that these screening tools resulted in an increased geriatric hearing screening rate or hearing aid use. There was also a global lack of improvement in hearing loss burden as measured by age-standardized disability-adjusted life years (DALYs) over 25 years (1990~2015) [26], implying that the available screening methods have not been accepted by the target facilities, including clinics with heavy flows of patients and densely populated communities. The barriers of cost, time and training still exist, leaving age-related hearing loss largely underdetected.

No parsimonious and feasible screening instrument to date has been developed to fill the above need. The present study was the first attempt to simplify the clinical standard pure tone audiometric assessment by using a machine learning technology on a large pure tone evaluation dataset collected from communities. The goal was to explore an efficient data-driven approach is applicable in the community-based physical check setting for screening moderate-to-profound hearing loss. Specifically, we aimed to implement decision tree algorithms to determine objectively the acoustical screening criteria for hearing classification.

Decision tree is a nonparametric supervised machine learning method used for classification and regression. It creates a practical model that classifies target conditions or predicts the value of a target variable by learning simple decision rules inferred from a set of training data features with a known output. The features most highly related to the outcome are included in the model. Decision tree methodology is ideal for building clinically useful classification models because it uses simple logic for classifying conditions, making it easy for patients and clinicians to interpret data [27–30].

The decision tree model, as a basic machine learning form, is playing an increasingly important role in healthcare applications. Specifically in the hearing science field, this technique has been used to seek the optimal suprathreshold test battery to classify auditory profiles towards effective hearing loss compensations [31]. More frequent applications of a decision tree analysis were evaluating whether a medical and audiological practice is cost-effective, for example, implanting cochlear prosthesis [32], the pursuit of magnetic resonance imaging (MRI) with or without contrast in the workup of undifferentiated asymmetrical sensorineural hearing loss [33], and universal or selective hearing screening on newborns [34, 35]. Some sophisticated machine learning technologies (e.g., neural network multilayer perceptron, support vector machine, random forest, adaptive boosting) have also been used in the hearing healthcare area, for instance, predicting postoperative monosyllabic word recognition performance in adult cochlear implant recipients [36] or predicting noise-induced hearing loss of manufacture workers based on demographic information and working acoustical environments [37, 38]. However, to the best of our knowledge, machine learning technologies have not yet been applied in the geriatric hearing screening area for the purpose of a practical implementation.

A decision tree analysis is commonly used to help identify a strategy most likely to reach a goal. In the context of the current study, a decision tree can produce a simple explicative model to determine which pure tone frequencies and intensities could maximize the screening test efficiency. A decision tree has proven a valuable tool for extracting meaningful information from measured data and represents a plausible solution for massive data learning tasks [39]. Additionally it has the advantages of a nonparametric setup, the tolerance of heterogeneous data, and the immunity to noise [40]. The detailed model deduction theory can be found in Breiman and Friedman [41], Friedman [42] and Quinlan [43]. In contrast, most advanced machine learning models answer “yes/no” questions by generating complex structural functions rather than specific values. For example, the neural networks models produce trained functions dependent on network topology and weighted factors [44]. A stochastic based method named Naive Bayes [45, 46] generates a statistical distribution network, which can hardly be parsed as a deterministic function. Although combining multiple decision trees in a random forest [47] may offer better prediction accuracies, given the small number of predictive features and the specific goal of developing explicit classification rules for hearing screening, we concluded that the decision tree method was sufficient to meet our needs.

The research question guiding the present study was whether there is a parsimonious pure tone set that can predict moderate-to-profound hearing loss based on the clinical standard audiometric pure tone thresholds measured in the community-dwelling geriatric population. The classification boundary of moderate hearing loss (pure tone average PTA > 40 dB HL) was selected because moderate or greater hearing loss in older adults is significantly associated with an increased risk of developing frailty [48], lower levels of physical activity [49], and a 20% increased risk of mortality after adjusting for demographics and cardiovascular risk factors [50]. More importantly, older adults with moderate-to-profound hearing loss benefit from hearing aids or cochlear implants not only in terms of improved hearing function but also in terms of positive effects on anxiety, depression, health status, and quality of life [51]. Our hypothesis was that a pair of tones with specific intensities calculated via decision tree analysis on the pure tone thresholds at four key frequencies (i.e., 500 Hz, 1 kHz, 2 kHz and 4 kHz) could be used to detect moderate-to-profound hearing loss with high (> 85%) accuracy, sensitivity and specificity.

## Methods

### Participants

Adults aged 60 years or older from two typical communities (with populations of approximately 110,000 and 84,000) in Shanghai were recruited to undertake clinical audiometric pure tone testing. No history of audiological rehabilitation experience (e.g., amplification, auditory training) by self-report was required to participate in the study. A total of 1322 participants were recruited from the Jiuting community (community A), yielding a final sample size of *n* = 1261 (mean age = 71.4 yrs., range 60–92) after excluding 61 participants due to missing audiometric data from either ear. Of the 536 participants recruited from the Nanmatou community (community B), 4 were excluded from the analysis due to missing audiometric data, yielding a final sample size of *n* = 532 (mean age = 76.5 yrs., range 60–104). The demographic information regarding age, sex and hearing severity are displayed in Table 1.

**Table 1:** Demographic Characteristics of Participants Aged 60 Years or Older With Audiometric Testing. The degree of hearing loss was classified based on the pure tone average thresholds (PTA) of the better and worse ear

	Community A (*n* = 1261)				Community B (*n* = 532)			
	Better-hearing ear		Worse-hearing ear		Better-hearing ear		Worse-hearing ear	
	NH (*n* = 18)	HL (*n* = 1243)	NH (*n* = 9)	HL (*n* = 1252)	NH (*n* = 84)	HL (*n* = 448)	NH (*n* = 33)	HL (*n* = 499)
Sex								
Male	6 (33.3%)	540 (43.4%)	5 (55.6%)	541 (43.2%)	30 (35.7%)	294 (34.4%)	8 (24.2%)	176 (35.3%)
Female	12 (66.7%)	703 (56.6%)	4 (44.4%)	711 (56.8%)	54 (64.3%)	154 (65.6%)	25 (75.8%)	323 (64.7%)
Age group								
60–69	8 (44.4%)	526 (42.3%)	4 (44.4%)	530 42.0%)	51 (60.7%)	118 (26.2%)	25 (75.8%)	144 (28.9%)
70–79	9 (50%)	609 (49%)	4 (44.4%)	614 (49.3%)	22 (26.2%)	127 (28.3%)	6 (18.2%)	143 (28.7%)
≥ 80	1 (5.6%)	108 (8.8%)	1 (11.2%)	108 (8.7%)	11 (13.1%)	203 (45.4%)	2 (6.0%)	212 (42.4%)
Hearing symmetry								
Symmetrical PTA	18 (100%)	1197 (96.3%)	9 (100%)	1206 (96.3%)	79 (94%)	402 (89.7%)	33 (100%)	447 (89.6%)
Asymmetrical PTA	0	46 (3.7%)	0	46 (3.7%)	5 (6%)	46 (10.3%)	0	52 (10.4%)
Hearing severity based on the WHO standard								
Mild (PTA 26~40 dB HL)		598 (47.4%)		360 (28.8%)		202 (38%)		169 (33.9%)
Moderate (PTA 41~70 dB HL)		576 (45.7%)		725 (57.9%)		176 (33.1%)		191 (38.3%)
Severe (PTA 71~90 dB HL)		61 (4.8%)		135 (10.8%)		60 (11.3%)		90 (18.0%)
Profound (PTA > 90 dB HL)		8 (0.6%)		32 (2.5%)		10 (1.9%)		49 (9.8%)

### Audiometric assessment

Audiometric pure tone testing was administered by two trained audiologists in a quiet consulting room in each of the community healthcare centers located within a 15-min walking distance from the residents’ homes. Prior to the audiometric assessment, the ears were examined for wax or abnormalities. The air conduction thresholds were obtained at 0.5~4 kHz over an intensity range of −10 to 120 dB using an Interacoustics MA52 audiometer with manual testing per protocol. Each individual’s hearing thresholds were annotated as dB HL at each of 4 frequencies (500 Hz, 1 kHz, 2 kHz and 4 kHz). Stimuli were presented through supra-aural headphones (TDH-39), except in rare cases of ear canal collapse or crossover retesting when insert earphones (Ear Tone 3A) were used. As acoustic isolation was not available, the ambient noise levels were monitored during the pure tone testing by an AWA5636 sound level meter. The average ambient noise level ranged between 35 and 38 dBA.

Hearing loss severity was defined per the classification of the World Health Organization as a pure tone threshold (PTA at 0.5, 1, 2 and 4 kHz) between 25 dB HL and 40 dB HL indicating mild hearing impairment and a hearing threshold of more than 40 dB HL indicating moderate or more severe hearing impairment. Data were collected over a 4-month period in community A and a 2.5-month period in community B.

### Analysis strategy

The descriptive statistics related to the community sample characteristics were evaluated using PASW 24 (SPSS/IBM, Chicago, IL). Pearson’s chi-square test was employed for the analysis of proportions comparison. Regression analysis was performed to test the relationship between age and hearing acuity. The level of significance was established at the 0.05 level.

The decision tree analysis was performed using the Python 3.7.0 SKlearn 0.19.2 package. We used a decision tree approach with a depth of 2 levels to analyze the audiometric pure tone data. A deeper tree was not considered in this study because the number of features in the model (i.e., 4 frequencies) associated with the hearing severity determination was fairly small, and the goal of the study was to simplify the hearing assessment procedure by using only 2 of the 4 frequencies. The known classification output for model training and testing was two classes of hearing status labeled as “normal-to-mild hearing loss (PTA<=40 dB HL)” and “moderate-to-profound hearing loss (PTA>40 dB HL)”. Data collected from community A (*n* = 1261, i.e., 70.3% of the dataset) served as the training dataset, and data collected from community B (*n* = 532, i.e., 29.7% of the total datasets) served as the test dataset in the decision tree analysis.

**Fig. 1:**
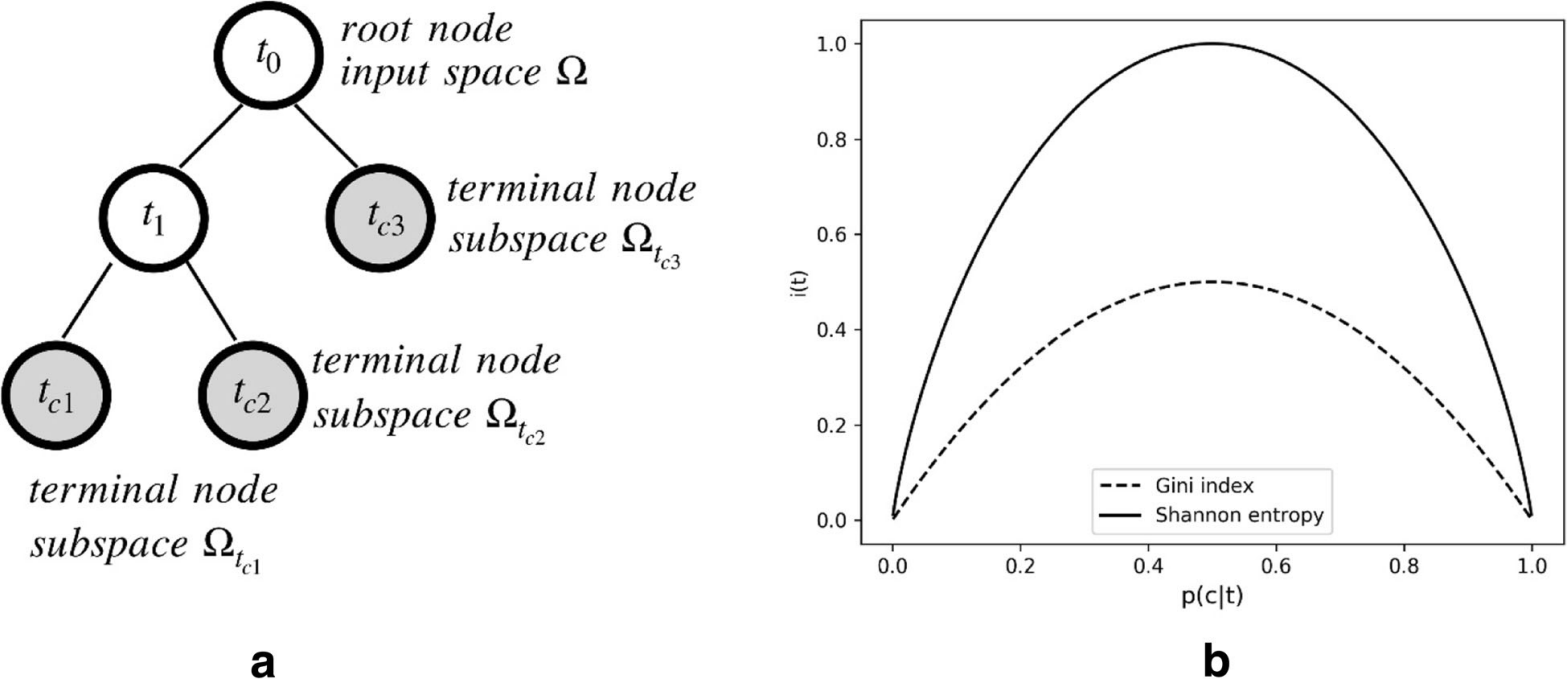
**a** Decision tree graph form, node definitions and space divisions. **b** Impurity function plotting. The solid line represents the Shannon entropy function, and the dashed line represents the Gini index

A brief mathematical illustration of the tree-based machine learning methodology used in the current study is displayed in Fig. 1. We defined the input variable (i.e., determinant pure tone frequency) as *X*_*i*_, where *X*_*i*_ ∈ Ω_*i*_ corresponds to the variable of the *i*-th input space. The ideal learning function is defined as *f* : *X* → *Y*, where *Y* ∈ {*c*_1_, *c*_2_, …, *c*_*n*_} is a finite set of labels for the classification problem. A tree-based model is a special representation of *f* with a rooted tree whose node *t* partitions the input space into the subspace Ω_*t*_, as shown in Fig. 1a. Ultimately, terminal nodes *t*_*ci*_ represent the best guess of $\hat{Y}\in \left\{\hat{c_1},\hat{c_2},\dots, \hat{c_n}\right\}$. The selection of the attribute used at each node of the tree to split the data is crucial for correct classification. Different split criteria (functions) were proposed in the literature [52], and we applied two widely used impurity functions in the study: Shannon entropy [53] and the Gini index [54]. Equations (1) and (2) quantified the uncertainty function *i*(*t*) with node *t* classification ratio $p\left({c}_k\mid t\right)={N}_{c_kt}/{N}_t$, where *N* is the number of samples.

1 $$ {i}_{Shannon}(t)=-\overset{m}{\sum \limits_{k=1}}p\left({c}_k\mid t\right) lo{g}_2\left(p\left({c}_k\mid t\right)\right) $$

2 $$ {i}_{Gini}(t)=\overset{m}{\sum \limits_{k=1}}p\left({c}_k\mid t\right)\left(1-p\left({c}_k\mid t\right)\right) $$

The above functions were plotted in Fig. 1b.

Conventionally in the clinical hearing severity classification, all 4 frequencies are assumed to be equally important and are required upon a hearing assessment when determining severity. To simplify the procedure for screening purposes, we computed the importance rank of the 4 frequencies through the tree structure training process, seeking to discover the 2 most important frequencies of the 4 for classification with high accuracy. The importance weight of each frequency was computed using Eq. (3) [41]:

3 $$ Importance={N}_t/N\ast \left(i(t)-{N}_{tr}/{N}_t\ast i\left({t}_r\right)-{N}_{tl}/{N}_t\ast i\left({t}_l\right)\right) $$

where *N* is the total number of samples, *N*_*t*_ is the number of samples for the current node, *N*_*tr*_ is the number of samples for the child node on the right side of the tree branch, *N*_*tl*_ is the number of samples for the child node on the left side of the tree branch, and *i* is the to-be-optimized impurity function for the corresponding nodes.

In addition to the decision tree analysis, 3 state-of-the-art machine learning analyses applicable to our datasets were performed for classification outcome comparisons. These machine learning models included Support Vector Machine (SVM), Random Forest (RF), and Multilayer Perceptron (MLP). The same training and testing data used in the decision tree were fed to these models. The SVM model was fitted with the Radial Basis Function kernel [55]. The RF was set up with a depth of 2 for each of 100 estimators [56]. MLP was constructed with 100 hidden layers, and the optimization solver was set as “adam” [57]. The analyses were implemented in Python with the Scikit-learn package [58].

As an individual’s hearing status can be labeled either by the better-hearing ear’s PTA according to WHO or based on the worse-hearing ear’s PTA for screening purposes as suggested by hearing screening researchers [22, 59], the decision tree analysis was performed on the pure tone thresholds of the better-hearing ear and the worse-hearing ear separately.

## Results

### Descriptive analysis

The demographic characteristics of the participants from the two communities are shown in Table 1. The two geriatric samples displayed a number of distinct characteristics. On average, participants from community A (M = 71.41, SD = 5.32) were significantly younger than participants from community B (M = 76.55, SD = 10.18), *t* (1791) = 13.962, *p* < .001. The sex ratio of participants with hearing loss showed opposite patterns between the two communities; there were significantly more male than female participants with hearing loss in community B, *λ*^2^(1)=9.303, *p* = .002. The hearing severity distribution was significantly different between the two community samples depending on how hearing loss was defined, *λ*^2^(4)=186.086, *p* < .01. Specifically, when hearing loss was defined by the better-hearing ear’s PTAs, there was no significant difference in the proportion of older people with moderate-to-profound hearing loss between community A (51.9%) and community B (54.9%%), *λ*^2^(1)=1.205, *p* = .272. However, if hearing severity was categorized according to the worse-hearing ear’s PTAs, the prevalence of moderate-to-profound hearing loss was significantly higher in community A (70.7%) than in community B (62.0%), *λ*^2^(1)=13.070, *p* < .01.

Asymmetrical PTA was defined in the present study as a difference in the four-frequency pure tone average between the worse- and better-hearing ears larger than 20 dB HL. The PTA difference between better- and worse-hearing ears ranged from 0 dB HL to 55 dB HL in the community A sample and from 0 dB HL to 68.75 dB HL in the community B sample. The community A geriatric sample (3.7%) had a significantly smaller proportion of asymmetrical PTA than the community B sample (10.4%), *λ*^2^(1)=30.738, *p* < .01.

The regression analysis on the PTA predicted by age illustrated that age was significantly associated with hearing acuity (*B* = .601, *F* (1,1791) = 256.418, *p* < .001, adjusted *R*^2^ = .125). Specifically, the hearing acuity dropped 0.6 dB for every one-year increase in age above 60 years old.

### Decision tree analysis

**Fig. 2:**
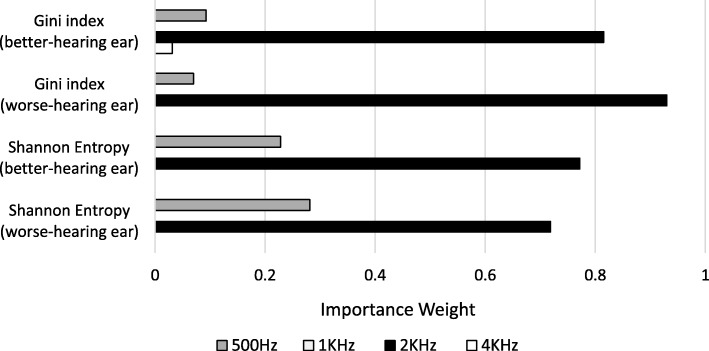
The importance weight of each determinant frequency as a result of the optimal Gini index function and the Shannon entropy function

The implemented decision tree analysis using two impurity functions generated the same results, namely, that the 0.5 kHz tone and 2 kHz tone were the two most important frequencies for classifying older adults with and without moderate-to-profound hearing loss (Fig. 2).

**Fig. 3:**
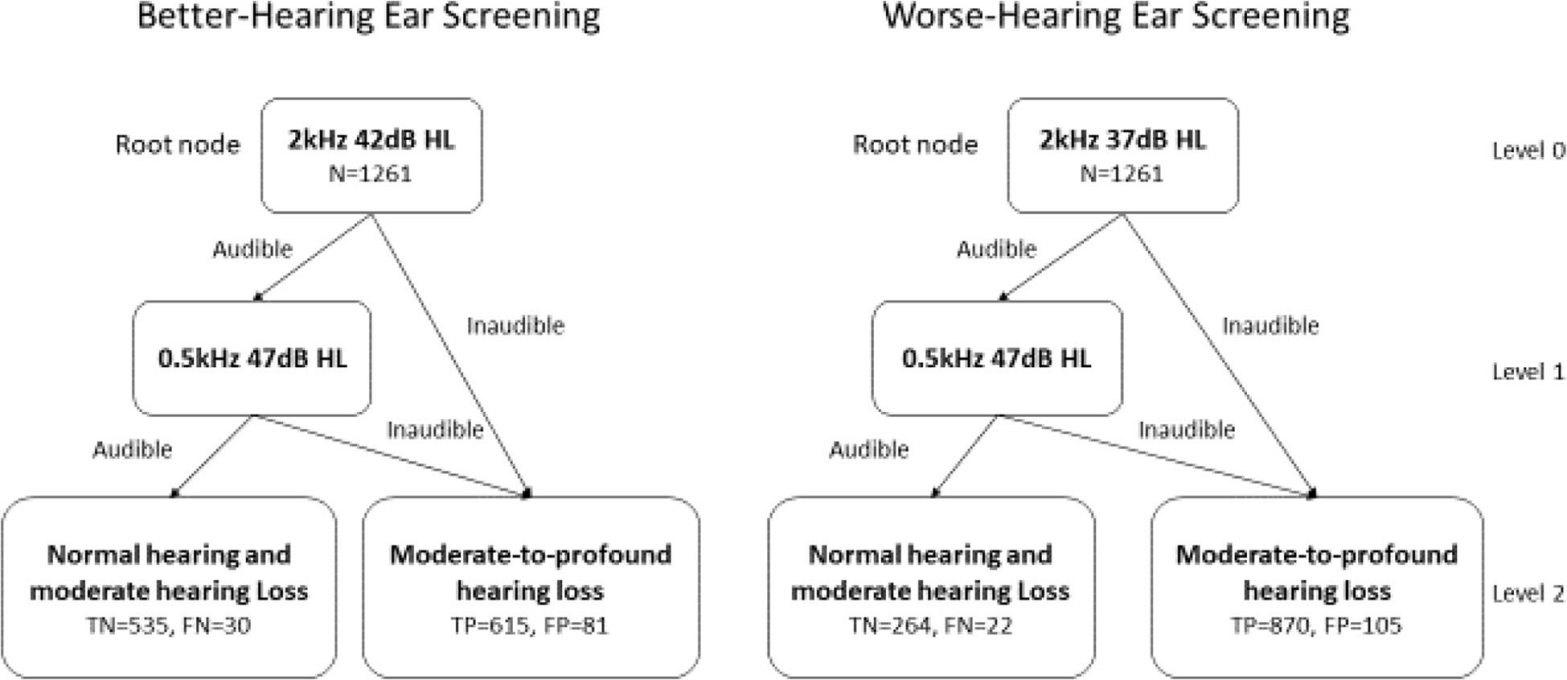
Decision trees generated using threshold data from the better-hearing ear (left panel) and worse-hearing ear (right panel). Classification results were indicated by false positives (FP), false negatives (FN), true positives (TP), and true negatives (TN)

The tree model suggested a simple two-step screening approach (Fig. 3): the 2 kHz tone with a specific intensity is presented at the first step, followed by a 0.5 kHz tone depending on the individual’s response to the 2 kHz tone. The screening tone intensities were determined based on the optimal Gini index and Shannon entropy. When an individual’s hearing severity was defined by the better-hearing ear, a combination of a 2 kHz (42 dB HL) tone and a 0.5 kHz (47 dB HL) tone was suggested. When the worse-hearing ear was selected to determine an individual’s hearing status, a combination of a 2 kHz (37 dB HL) tone and a 0.5 kHz (47 dB HL) tone was suggested.

The performance of the decision tree analysis for moderate-to-profound hearing loss prediction was evaluated in terms of sensitivity, specificity and accuracy calculated from the confusion matrices summarized in Table 2. The confusion matrices present the detailed predictions produced from the datasets at the optimal threshold based on the Gini index (the Shannon entropy produced the same results). The sensitivity, specificity and accuracy are displayed in Table 3. The decision tree for the better-hearing ear screening achieved a classification accuracy of 91.20% with a sensitivity of 96.35% and a specificity of 86.85% based on the training dataset and obtained a consistent classification outcome when tested with the community B dataset. The decision tree for the worse-hearing ear screening achieved a classification accuracy of 89.93% with a sensitivity of 97.53% and a specificity of 71.54% for the training dataset and obtained a comparable classification outcome when tested with the community B dataset. The participants who were misclassified as having moderate-to-profound hearing loss (false positives) all had mild hearing loss.

**Table 2:** Confusion matrix summarizing the number of false positives (FP), false negatives (FN), true positives (TP), and true negatives (TN). Numbers in parentheses are the count from the test dataset (community B)

		Actual (better-hearing ear)		Actual (worse- hearing ear)	
		Positive	Negative	Positive	Negative
Predicted	True	615 (230)	535 (259)	870 (322)	264 (163)
	False	30 (27)	81 (16)	22 (8)	105 (39)

**Table 3:** Sensitivity, specificity and accuracy of classifying moderate-to-profound hearing loss by the computed screening tones

	Better-hearing ear		Worse-hearing ear	
	Training Set (Community A)	Test Set (Community B)	Training Set (Community A)	Test Set (Community B)
Sensitivity	95.35%	93.50%	97.53%	97.58%
Specificity	86.85%	90.56%	71.54%	80.69%
Accuracy	91.20%	91.92%	89.93%	91.17%

The classification performance of the advanced machine learning methods were displayed alongside with the decision tree outcomes in Table 4. The results suggested that the outcomes of the decision tree approach were comparable with the random forest. Both the decision tree and the random forest were superior to the support vector machine and multilayer perceptron in the current study when comprehensively considering the sensitivity, specificity and accuracy in the test set.

**Table 4:** Comparisons on the sensitivity, specificity and accuracy of classification among different machine learning approaches. Performances greater than 85% are highlighted in bold

			Sensitivity	Specificity	Accuracy
Better-hearing ear	Training set (Community A)	Decision Tree (DT)	**95.35%**	**86.85%**	**91.20%**
		Support Vector Machine (SVM)	**100.00%**	**100.00%**	**100.00%**
		Random Forest (RF)	**91.32%**	**94.97%**	**93.10%**
		Multilayer Perceptron (MLP)	78.29%	56.98%	67.88%
	Test set (Community B)	Decision Tree (DT)	**93.50%**	**90.56%**	**91.92%**
		Support Vector Machine (SVM)	**100.00%**	33.57%	64.29%
		Random Forest (RF)	**94.31%**	**92.31%**	**93.23%**
		Multilayer Perceptron (MLP)	**90.65%**	51.05%	69.36%
Worse-hearing ear	Training set (Community A)	Decision Tree (DT)	**97.53%**	71.54%	**89.93%**
		Support Vector Machine (SVM)	**100.00%**	**100.00%**	**100.00%**
		Random Forest (RF)	**98.21%**	77.51%	**92.15%**
		Multilayer Perceptron (MLP)	**99.89%**	11.38%	73.99%
	Test set (Community B)	Decision Tree (DT)	**97.58%**	80.69%	**91.17%**
		Support Vector Machine (SVM)	**100.00%**	34.16%	75.00%
		Random Forest (RF)	**99.39%**	84.65%	**93.80%**
		Multilayer Perceptron (MLP)	**100.00%**	19.31%	69.36%

**Fig. 4:**
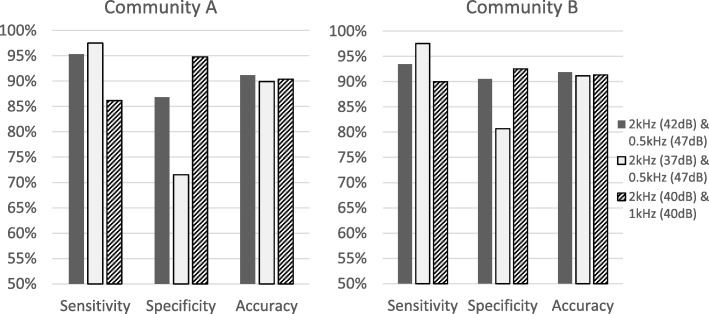
The sensitivity, specificity and accuracy as a result of different screening approaches. The left panel displays the classification performance using community A’s data, and the right panel displays the classification performance using community B’s data

In addition, the intuitive screening criteria of 40 dB HL at 2 kHz and 1 kHz suggested by previous literature were tested in our sample as a comparison. The confusion matrix for classifying moderate-to-profound hearing loss in community A indicated a TP of 556, TN of 584, FP of 32 and FN of 89, resulting in an accuracy of 90.4% (with a sensitivity of 86.20% and a specificity of 94.81%). The classification using 40 dB HL cutoffs in community B produced an accuracy of 91.35% (with a sensitivity of 90.00% and a specificity of 92.55%). The performance of different screening criteria is illustrated in Fig. 4.

## Discussion

The current study used a fundamental machine learning technique to explore a parsimonious pair of pure tones that can be applied for hearing screening with a sensitivity, specificity and accuracy over 85% in community-dwelling older adults over 60 years of age. This was the first effort to objectively simplify the standard clinical pure tone hearing test procedure and construct an acoustic hearing screening criteria feasible for large-scale geriatric hearing screening. The screening tones were determined by collecting standard audiometric testing results from 1793 older community residents and by implementing decision tree analysis on their pure tone thresholds.

In line with Ciurlia-Guy et al.’s [60] finding, our decision tree analysis identified 2 kHz as the most important frequency for moderate-to-profound hearing loss screening in elderly people (Fig. 2). However, distinct from the conventional belief in the important role of 1 kHz in hearing screening, our results indicated that 1 kHz had a minimal impact on hearing classification at the moderate severity level; instead, 0.5 kHz became the second most important frequency. The tree model suggested using a 2 kHz tone coupled with a 0.5 kHz tone to achieve the optimal classification performance. The most appropriate intensities of the screening tones quantified based on the Gini index for the better-hearing ear were 42 dB HL at 2 kHz and 47 dB HL at 0.5 kHz. This procedure increased the sensitivity and accuracy by 9.1 and 1.0%, respectively, compared to using the intuitive 40 dB HL at 2 kHz and 1 kHz as the criteria (Fig. 4). Sensitivity is important in the context of this study because early intervention for as many individuals with true moderate or greater hearing loss as possible is desired. On the other hand, specificity also needs to be accounted for to minimize the unnecessary number of false referrals. The improvement of sensitivity by using 42 dB HL at 2 kHz is clinically significant when the screening target is a large geriatric population.

The optimal screening criteria computed based on the worse-hearing ear were slightly different at 2 kHz due to the proportion of participants with asymmetrical hearing loss. The cutoff value of 37 dB HL produced a sensitivity of 97.53% with an accuracy of 89.93%, which implies a more aggressive screening approach. However, the specificity was compromised by 15.31%, which indicates a high false positive rate that will result in a waste of health resources. The difference in the criteria between the two ears suggests a consideration of what rationale to follow prior to massive screening. If healthcare providers believe that older adults’ hearing severity should be defined by the worse ear’s PTA and that unilateral hearing loss should be treated in this population, then the criteria computed based on the worse-hearing ear thresholds are recommended. If healthcare providers support that the better ear represents an individual’s overall hearing function and an early intervention should be implemented when the better ear has moderate or greater hearing loss, then the criteria calculated based on the better-hearing ear thresholds are recommended.

The results of the current study suggested that the decision tree models constitute useful analytical tools to screen moderate-to-profound hearing loss. The decision tree technique formed a simple explicative model and produced easy-to-interpret results (Fig. 3). Although the random forest produced slightly higher classification sensitivity, specificity and accuracy in the current study, it did not provide an explicit cutoff criteria for practical use. Other advanced machine learning techniques, such as support vector, were able to “learn” considerably well from the training set; however, the trained models did not effectively generalize to new datasets (Table 4), which was reflected by the poor specificities (< 55%) compromising the overall accuracy (< 80%). Those state-of-the-art methods did not deduce a cutoff criteria to meet the purpose of the current study either. Compared to those advanced black-box-based machine learning algorithms, the decision tree method demonstrated the advantage of knowledge extraction that requires explicit explanations of the data relationships.

The results can be readily translated into feasible clinical applications. In the simplest form, an inexpensive well-calibrated sound generator can be used to incorporate the computed pure tones and deliver them in the correct order to the target individuals. This method integrates the advantages of previous screening approaches. First, its accuracy is as high as that of the screening audiometers reported in the literature (e.g., AudioScope). In both community samples, the accuracy of using the pair of 2 kHz and 0.5 kHz tones exceeded 91%. Second, since the screening criteria were directly calculated based on the standard pure tone thresholds, the correlation of the screening result with the gold standard audiometric results is inherently strong. Third, screening with acoustically calibrated tones is perceived as more objective than using self-report and uncalibrated methods such as the whisper test [61] by both clinicians and patients and consequently will motivate greater engagement of healthcare providers from multidisciplinary areas in the hearing screening program. Fourth, the simple two-step procedure can save substantial time for clinicians. Screening results can be gathered in less than 1 min (including delivering the 4 tones (2 to each ear) and receiving responses) for each individual. No specialized training is needed, and any healthcare provider is able to determine whether to make a referral for further diagnostic audiometric evaluation and treatment by following the tree model. Finally, this method can be cost-effective. Compared to the currently available screening devices, which are relatively expensive, a sound generator programmed with precise screening tones will be more affordable for health centers at the community level.

The prevalence of moderate-to-profound hearing loss shown in this study’s samples (52 and 60% in communities A and B, respectively) was consistent with the prevalence documented in previous epidemiological studies [23, 62–64] (Table 1). The proportion of older adults with normal hearing was low due to the testing conditions in a quiet but nonsoundproofed room. A portion of the participants with normal hearing were likely to be misdiagnosed as having mild hearing loss, but this had a minimum impact on our results because the classification boundary was set at moderate hearing loss (PTA = 40 dB HL). The ambient noise level in the test environment was under 40 dBA, which permitted the accurate evaluation of the moderate-to-profound pure tone thresholds. Asymmetrical hearing loss was not common in our study samples, which is consistent with the literature [65]. The significant positive linear relationship between hearing loss and age found in our study is also in line with previous studies [17, 62]. Participants in the study were not screened for family history of deafness, dementia or occupational noise exposure, and we believe that they are representative of the general elderly population. Although the age, sex, hearing loss severity and asymmetry distribution characteristics were different between the two community samples, the decision tree model generated by analyzing the data from community A was robust. The tree model resulted in comparable classification performance when tested on the data of community B.

The results of the present study illustrated that hearing loss is largely underrecognized in the community-dwelling geriatric population. Given the increasing prevalence and disease burden of undetected hearing loss in older adults and the availability of effective treatment (e.g., modern amplification devices, aural rehabilitation), early identification of individuals with moderate or greater hearing loss is a cost-saving strategy. However, screening methods at the community level are inadequate. There is an unmet need for a feasible data-driven screening protocol for healthcare policy makers to promote early identification and immediate treatment of hearing loss in geriatric patients. The current study demonstrated how a machine learning technique, specifically decision tree analysis, can be used in the hearing healthcare setting to meet the need for a parsimonious acoustical screening protocol for community-dwelling geriatric populations. Community health care centers have the potential to serve as the first point of access to identify moderate or greater hearing loss. The proposed simple two-step pure tone screening approach made it possible for the hearing test to be included in routine checkups. This study suggests a feasible and powerful means to deliver quality services when the gold standard audiometric evaluation of hearing is not available.

The main limitation of our study was that our data were collected from two communities in a single city, which may affect the generalizability of the results. Validating the tree model with audiometric data from communities in a few randomly selected cities and rural areas is needed to generalize the proposed screening instrument nationwide.

## Conclusion

Our study proposed a parsimonious two-step screening procedure using decision tree analysis. A pair of tones (2 kHz and 0.5 kHz) was identified to screen for moderate-to-profound hearing loss in community-based geriatric populations over 60 years of age in Shanghai. Implanting the pair of tones into a well-calibrated sound generator can create a simple, practical and time-efficient screening tool with high accuracy that can be made available at health centers at all levels and can thus facilitate the initiation of nationwide extensive hearing screening in older adults. The decision tree approach is an appropriate method to determine the optimal pure tone hearing screening criteria for local geriatric populations and identify high-risk subpopulations that need early prevention and intervention programs and, therefore, could be used to make the most of public health resources.

## Data Availability

The datasets used and analyzed during the current study are available from the corresponding author upon reasonable request.

## Abbreviations

dB HL: Decibel hearing level
PTA: Pure tone average
